# Discrepancies Between Predicted and Measured Microelements and Toxic Elements in Beetroot Supplemented Diets

**DOI:** 10.3390/toxics14080721

**Published:** 2026-08-14

**Authors:** Michał S. Majewski, Łukasz Smyk, Anetta Hanć, Agnieszka Owczarczyk-Saczonek, Waldemar Jarosław Grzegorzewski, Joanna Majkowska-Gadomska, Anna Francke

**Affiliations:** 1 Department of Pharmacology and Toxicology, Faculty of Medicine, University of Warmia and Mazury, 10-082 Olsztyn, Poland; lukasz.smyk@gmail.com; 2 Department of Trace Analysis, Faculty of Chemistry, Adam Mickiewicz University, 61-614 Poznań, Poland; anetta.hanc@amu.edu.pl; 3 Department and Clinic of Dermatology, Sexually Transmitted Diseases and Clinical Immunology, University of Warmia and Mazury, Al. Wojska Polskiego 30, 10-959 Olsztyn, Poland; 4 Faculty of Biology, Nature Protection and Sustainable Development, University of Rzeszów, 35-310 Rzeszów, Poland; wgrzegorzewski@ur.edu.pl; 5 Department of Agroecosystems and Horticulture, Faculty of Agriculture and Forestry, University of Warmia and Mazury, 10-718 Olsztyn, Poland; majkowska-gadomska@uwm.edu.pl (J.M.-G.); afrancke@uwm.edu.pl (A.F.)

**Keywords:** trace elements, toxic elements, additive modelling, *Beta vulgaris*, ICP-MS, elemental analysis

## Abstract

**Background**: Accurate assessment of microelement and toxic element content in animal diets is essential for diet formulation, quality control, and exposure assessment; however, predictive additive models may not fully account for variability arising from raw materials and processing factors. **Methods**: This study evaluated the composition of microelements and toxic elements in the four diets enriched with 4% beetroot cultivars *Boldor* and *Wodan*, either without (level 1) or with (level 3) foliar application of the selenium–based plant growth stimulator. Predicted values (calculated using an additive model *P* = 0.96 × *C _basal diet_* + 0.04 × *C _beetroot component_*) were compared with experimentally measured concentrations (M). Four dietary variants *Boldor* 1, *Boldor* 3, *Wodan* 1 and *Wodan* 3 were analyzed. Elemental concentrations of Fe, Zn, Cu, Cr, Ni, Se, Pb, As, Cd, and Sb were determined using ICP-MS. **Results**: Substantial discrepancies between predicted (P) and measured (M) values were observed. The largest deviations occurred for Cr, Fe, and Sb. Cr and Sb were generally underestimated by the model, whereas Fe was consistently overestimated. Selenium (Se) showed moderate variability, while Cu, Zn, and Ni exhibited close agreement between predicted and measured values. The largest measured cultivar-related differences were observed for As (2.87-fold), Cd (2.78-fold), Pb (2.47-fold), Cr (2.37-fold), Fe (1.73-fold), Sb (1.57-fold), Cu (1.13-fold), and Zn (1.05-fold), all of which occurred at higher concentrations in *Wodan* than in *Boldor*. No statistically significant effect of the selenium–based plant growth stimulator was observed. **Conclusions**: Overall, the results demonstrate that additive models have limited accuracy in predicting trace element composition in complex diets, particularly for elements potentially affected by raw-material variability, sampling heterogeneity, and analytical uncertainty. The contribution of the dietary 4% beetroot component played a key role in shaping element distribution, particularly for elements with high component-to-basal diet ratios. These findings highlight the importance of proper mixing, prevention of segregation in powdered diets, and strict control of raw material quality.

## 1. Introduction

The accurate determination of microelements and toxic elements in food and feed matrices is essential for assessing nutritional quality, safety, and compliance with regulatory standards. Trace elements such as iron (Fe), zinc (Zn), and copper (Cu) play key roles in physiological processes, whereas toxic elements including arsenic (As), cadmium (Cd), and lead (Pb) pose significant health risks even at low concentrations [1,2,3,4,5,6,7,8]. Accurate quantification and prediction of these elements in complex dietary systems remain critical challenges in food and nutrition research. The term “potentially toxic elements (PTEs)” is widely used in the environmental and food safety literature. However, given the ongoing debate regarding its scientific precision and the argument that the qualifier “potentially” may unnecessarily obscure the established toxicity of certain elements (e.g., Cd, Pb, Hg), this manuscript uses the term “toxic elements” throughout for elements with well-documented toxicological significance [9,10].

In feed formulation, additive models are commonly used to estimate the final composition of diets based on the proportional contribution of individual components. These models assume that the concentration of each element in the final mixture is a linear function of its content in the raw materials. While such an approach is convenient and widely applied, it does not account for potential interactions between components, variability in raw material composition, or technological factors affecting the distribution of elements [5,11]. As a result, discrepancies between predicted and measured values are frequently reported, particularly for trace elements present at low concentrations [12,13]. In the present study, a linear additive model (mass-balance model) was applied to estimate elemental concentrations in experimental diets according to the proportional contribution of the basal diet and the beetroot component. This model served as the theoretical reference for comparison with experimentally measured values.

Plant-based ingredients, including beetroot (*Beta vulgaris* L.), are increasingly incorporated into diets due to their nutritional value and bioactive compounds [14,15,16,17,18,19]. Previous studies have demonstrated considerable variability in the elemental composition of beetroot and red beet-based products [20,21,22,23] as well as the presence of toxic elements such as arsenic (As) and cadmium (Cd), highlighting potential health risks and the need for stricter quality control [24]. Plants are also known to accumulate microelements and toxic elements from soil, which may significantly influence the mineral composition of derived food or feed products [25,26,27]. The extent of this accumulation depends on multiple factors, including soil characteristics, environmental conditions, and agricultural practices. Consequently, the use of plant-derived components introduces an additional heterogeneity that may not be fully captured by predictive models.

Chronic exposure to toxic elements such as arsenic (As), cadmium (Cd), and lead (Pb) may adversely affect animal health through different toxicological mechanisms. Arsenic has been associated with oxidative stress and hepatic, renal, neurological, and cardiovascular dysfunction, whereas cadmium primarily accumulates in the kidneys and liver. Lead exposure may impair hematopoietic, nervous, renal, and cardiovascular function. Consequently, precise determination of toxic element concentrations in experimental diets is essential for accurate exposure assessment and interpretation of experimental results.

Another important, yet often overlooked, factor is the physical form of the diet and its influence on sample homogeneity. Powdered diets are particularly susceptible to incomplete mixing and particle segregation due to differences in particle size, density, and shape [25]. These processes may lead to non-uniform distribution of elements within the material, resulting in non-representative sampling and increased variation. Such effects are especially relevant for trace elements, where small differences in distribution can lead to substantial relative deviations.

In addition to raw material and technological factors, agronomic practices may also influence the elemental composition of plant-based ingredients. Plant treatments aimed at modifying nutrient uptake and stress responses have been proposed as tools to regulate the accumulation of trace elements [28,29]. However, the effectiveness and consistency of such approaches remain insufficiently understood, especially when evaluated at the level of final diet composition.

Therefore, the aim of the present study was to evaluate the differences between predicted and measured concentrations of selected microelements and toxic elements in four diets supplemented with two beetroot cultivars (*Boldor* and *Wodan*). As a secondary factor, we also examined whether foliar treatment with a selenium–based plant growth stimulator influenced the observed discrepancies. By identifying the key factors influencing deviations between theoretical and experimental values, this work provides insights into the limitations of additive models and highlights methodological considerations relevant to nutritional and feed analysis.

## 2. Materials and Methods

**Study Design**

The experiment was conducted as a two-factor study.

The first factor was the foliar application of the biostimulant. Two treatment levels were applied:

(1) control—plants sprayed with water only;

(2) biostimulant—3.0 L/ha in 300 L water.

The second factor included two beetroot cultivars differing in root coloration:

(i) Boldor—yellow-orange roots with smooth, round shape and high uniformity;

(ii) Wodan—standard, red-colored cultivar.

The field experiment was conducted using four independent plots for each treatment. Beetroot roots harvested from each plot were processed and analyzed separately, providing four independent biological replicates per treatment. Three analytical subsamples were prepared from each biological replicate, and their mean was used as the value for that replicate. Approximately 0.5000 g of each subsample was transferred into Teflon digestion vessels and subjected to independent microwave-assisted acid digestion. Each digest was subsequently analyzed separately by inductively coupled plasma mass spectrometry (ICP-MS) equipped with an octopole reaction system operated in helium collision mode. The analytical results obtained from the three independently prepared digests were averaged to obtain one representative value for each composite sample, and these values were used for statistical analyses.

**Selenium–Based Plant Growth Stimulator**

The formulation is intended for foliar application across various crop species to enhance tolerance to environmental and physiological stress, stimulate metabolic activity at the cellular level, and promote plant growth and development. According to the manufacturer (Arkop, Bukowno, Poland), the product is supplied as a liquid suspension with the following composition: Se 0.14% (1400 mg/kg), total amino acids 5.5%, nitrogen (N) 0.93%, and organic carbon 5%. The pH of the formulation ranges between 4.0 and 5.0.

For field application, the working solution was prepared at a volume of 300 L/ha, consisting of 3.0 L of selenium–based plant growth stimulator diluted in 297.0 L of water.

Selenium has been widely recognized as a beneficial element that may improve plant growth, antioxidant capacity, and tolerance to abiotic stress, although its effects depend on dose, plant species, and environmental conditions [30].

**Field Experiment**

The field trial was carried out in 2024 at the Didactic and Experimental Station of the University of Warmia and Mazury in Olsztyn, Poland (53.753569° N, 20.455521° E), on brown soil classified as quality class IIIb. Standard agronomic practices for table beet cultivation were applied throughout the growing season.

The experiment followed a randomized complete block design with four replicates per treatment to minimize spatial variability. Each experimental plot had an area of 10 m^2^ and was separated by buffer zones to prevent cross-contamination during spraying. Foliar applications were performed using a fine mist sprayer under favorable environmental conditions (low wind, moderate temperature), typically in the early morning or evening to ensure optimal uptake.

Seeds of the two beetroot cultivars (*Boldor* and *Wodan*) were sown in May 2024, and the roots were harvested in early October 2024. Foliar applications were performed at BBCH 31 (beginning of row closure, approximately 10% soil coverage by leaves), BBCH 37 (approximately 70% soil coverage), and BBCH 41 (beginning of storage root enlargement, roots > 2 cm in diameter), according to the standardized BBCH phenological scale. Foliar application was chosen because selenium uptake through the leaves is generally more efficient and less affected by soil physicochemical properties than root uptake. Moreover, this method allows precise control of the applied dose while minimizing variability associated with soil heterogeneity, thereby ensuring uniform and reproducible treatment conditions.

No symptoms of disease or pest infestation were observed during the experiment, and weed control was carried out manually.

Harvesting was performed at full physiological maturity during the first ten days of October, when roots reached a diameter of 5–8 cm and foliage showed signs of senescence. The total beetroot yield harvested from each experimental plot was approximately 8 kg. The harvested roots were thoroughly washed to remove adhering soil and subsequently dried at 65 °C in a laboratory oven (Binder ED400, Binder GmbH, Tuttlingen, Germany) until constant weight was achieved. Dried samples were subsequently homogenized using a knife mill (Grindomix GM300, Retsch GmbH, Haan, Germany) to obtain a fine powder for chemical analysis.

**Dietary Composition**

Experimental diets were prepared from individual ingredients, as presented in Table 1. A 4% (*w*/*w*) dried component (beetroot *Boldor* or *Wodan*) was incorporated into the diet. The diets were stored at −40 °C and thawed weekly. After thawing, the dried beetroot powder was passed through a 1 mm stainless-steel sieve to eliminate agglomerates and improve powder flowability before mixing. The sieved beetroot powder (4%, *w*/*w*) was then incorporated into the basal diet by replacing an equivalent proportion of corn starch and manually blended until no visible inhomogeneity was observed. The prepared diets were subsequently stored at 4 °C in sealed containers until administration to the rats.

The 4% dietary inclusion level was selected to ensure a measurable contribution of beetroot-derived trace elements while preserving the nutritional balance and physical characteristics of the experimental diets. In addition, this supplementation level was designed to approximate a realistic human dietary exposure, corresponding to the intake of approximately one medium-sized beetroot, thereby enhancing the translational relevance of the study.

Predicted elemental concentrations (P) in the experimental diets were calculated using a linear additive model according to the equation, see Table S1:

*P* = 0.96 × *C*_basal diet_ + 0.04 × *C*_beetroot component_ (1)

where P is the predicted concentration of a given element in the final diet, *C _basal diet_* is the concentration in the basal diet, and *C _beetroot_* is the concentration in the beetroot component. Both *C* _basal diet_ and *C* _beetroot component_ were determined experimentally using ICP-MS. Elemental concentrations of the basal diet and beetroot components used for the calculations are provided in Table S2 and Table S3, respectively. The model assumes complete homogeneity of the mixture and proportional contribution of each component to the final elemental composition.

**Sample Preparation and Microwave Digestion**

Prior to use, all Teflon vessels were acid-cleaned using the microwave-assisted cleaning procedure with 30% (*v*/*v*) HNO_3_ (65% HNO_3_, Suprapur, Merck, Darmstadt, Germany) for 0.5 h and rinsed twice with ultrapure water to prevent cross-contamination. Approximately 0.5000 g of each dried and homogenized sample was weighed and transferred into Teflon vessels of a microwave digestion system (Ethos One, Milestone, Sorisole, Italy). Samples were treated with 5 mL of ultrapure nitric acid (65% HNO_3_) and 1 mL of hydrogen peroxide (30% H_2_O_2_, TraceSELECT, Sigma-Aldrich, St. Louis, MO, USA).

The digestion procedure consisted of four stages:

(1) pre-digestion at room temperature for 3 h,

(2) heating to 200 °C with a holding time of 20 min at 1000 W,

(3) further heating to 220 °C with a holding time of 40 min at 1200 W,

(4) controlled cooling to 80 °C over 30 min.

After digestion, the solutions were diluted with ultrapure water. The dilution factor was 200, and final elemental concentrations were calculated on a dry weight (DW) basis. Procedural blanks were prepared and processed under identical conditions to monitor potential contamination.

**ICP-MS Analysis**

Elemental concentrations of Fe, Zn, Cu, Cr, Ni, Se, Pb, As, Cd and Sb were determined using an ICP-MS system (Agilent 7700×, Agilent Technologies, Tokyo, Japan) equipped with a quadrupole analyzer and an Octopole Reaction System (ORS), see Table 2. The ORS was operated in helium collision mode to minimize polyatomic interferences. ICP-MS is currently considered one of the most sensitive and reliable techniques for multi-element determination in complex food, feed and dietary supplement matrices, allowing simultaneous quantification of trace and ultra-trace elements with low detection limits and high analytical throughput [31].

Instrument parameters were optimized daily and maintained constant throughout the analysis (Table 2). High-purity argon (99.999%) was used as both the plasma and carrier gas. Rhodium (20 µg/L) was used as the internal standard because it is absent from the analyzed samples and provides effective correction for instrumental signal drift and matrix effects. Polyatomic interferences affecting Fe, Cr, As, and Se (e.g., ^40^Ar^16^O^+^, ^40^Ar^12^C^+^, ^40^Ar^35^Cl^+^) were minimized using the octopole reaction system (ORS) operated in helium collision mode. Residual spectral interferences were corrected mathematically using the following equation: Mc (82) = 1.0000 × M(82) − 1.0078 × M(83).

Calibration curves were established using external calibration with matrix-matched multi-element standards (Calibration Std 3., PerkinElmer, Waltham, MA, USA) prepared in 3.25% (*v*/*v*) HNO_3_. Calibration was performed over the concentration ranges of 1–500 µg/L for Fe and 0.05–100 µg/L for the remaining elements. All calibration curves demonstrated excellent linearity (R^2^ > 0.9996). Elemental concentrations were calculated and are reported on a dry weight (DW) basis following the sample drying procedure described above. Final concentrations are expressed as mg/kg DW. The analytical validation parameters applied in the present study are consistent with recommendations for trace element determination in dietary supplements and food matrices [31].

**Quality Control and Method Validation**

Analytical accuracy was verified using the certified reference material (CRM) NIST SRM 1570a (Trace Elements in Spinach Leaves), which was processed and analyzed under the same conditions as the samples. CRM measurements were performed in triplicate (*n* = 3). The comparison between certified and measured values is presented in Table S4, with recoveries ranging from 93.9% to 105.9%, confirming the accuracy of the analytical procedure. For Cr, Fe, and Sb, which are not certified in SRM 1570a, analytical accuracy was assessed using the standard addition method. Instrumental repeatability was assessed from three consecutive measurements of each digest and expressed as the coefficient of variation. Method detection limit (MDL) was calculated as 3.3 standard deviations of procedural blanks that underwent the same digestion procedure as the analyzed samples and ranged from 0.006 mg/kg for Cd to 0.5 mg/kg for Fe.

**Statistics**

Data are presented as mean ± standard deviation (SD). For each element, the agreement between predicted (P) and measured (M) concentrations was evaluated using the measured-to-predicted ratio (M/P) and the relative difference *(M − P)/P (%).*

The effects of beetroot cultivar (*Boldor* vs. *Wodan*; factor A), growth stimulator treatment (level 1 vs. level 3; factor B), and their interaction (A × B) on measured elemental concentrations were assessed using two-way analysis of variance (ANOVA). Cultivar and treatment were included as fixed factors, and the significance of the main effects (A and B) as well as the interaction effect (A × B) was evaluated.

Assumptions of normality and homogeneity of variance were verified using the Shapiro–Wilk and Levene’s tests. All datasets met the assumptions of normality and homogeneity of variance; therefore, two-way ANOVA was applied. Potential outliers were evaluated using graphical inspection and statistical assessment. No observations fulfilled the criteria for exclusion; therefore, all analytical results were retained in the final dataset and included in the statistical analyses. To control for multiple testing, *p*-values were adjusted using the Holm–Bonferroni procedure. Adjusted *p*-values < 0.05 were considered statistically significant.

## 3. Results

The largest measured cultivar-related differences were observed for As (2.87-fold), Cd (2.78-fold), Pb (2.47-fold), Cr (2.37-fold), Fe (1.73-fold), Sb (1.57-fold), Cu (1.13-fold), and Zn (1.05-fold), all of which occurred at higher concentrations in *Wodan* than in *Boldor*. These differences reflected the elemental composition of the beetroot roots, in which *Wodan* contained significantly higher concentrations of As, Cd, Pb, Cr, Fe, and Sb than *Boldor* (Table 3 and Table S3).

No statistically significant effect of the selenium-based foliar treatment (factor B) was detected for any of the analyzed elements (all *p* > 0.05; Table 4). Likewise, no significant cultivar × treatment interactions were observed (all *p* > 0.05), indicating that the foliar application did not consistently influence the elemental composition of the experimental diets.

**Figure 1:**
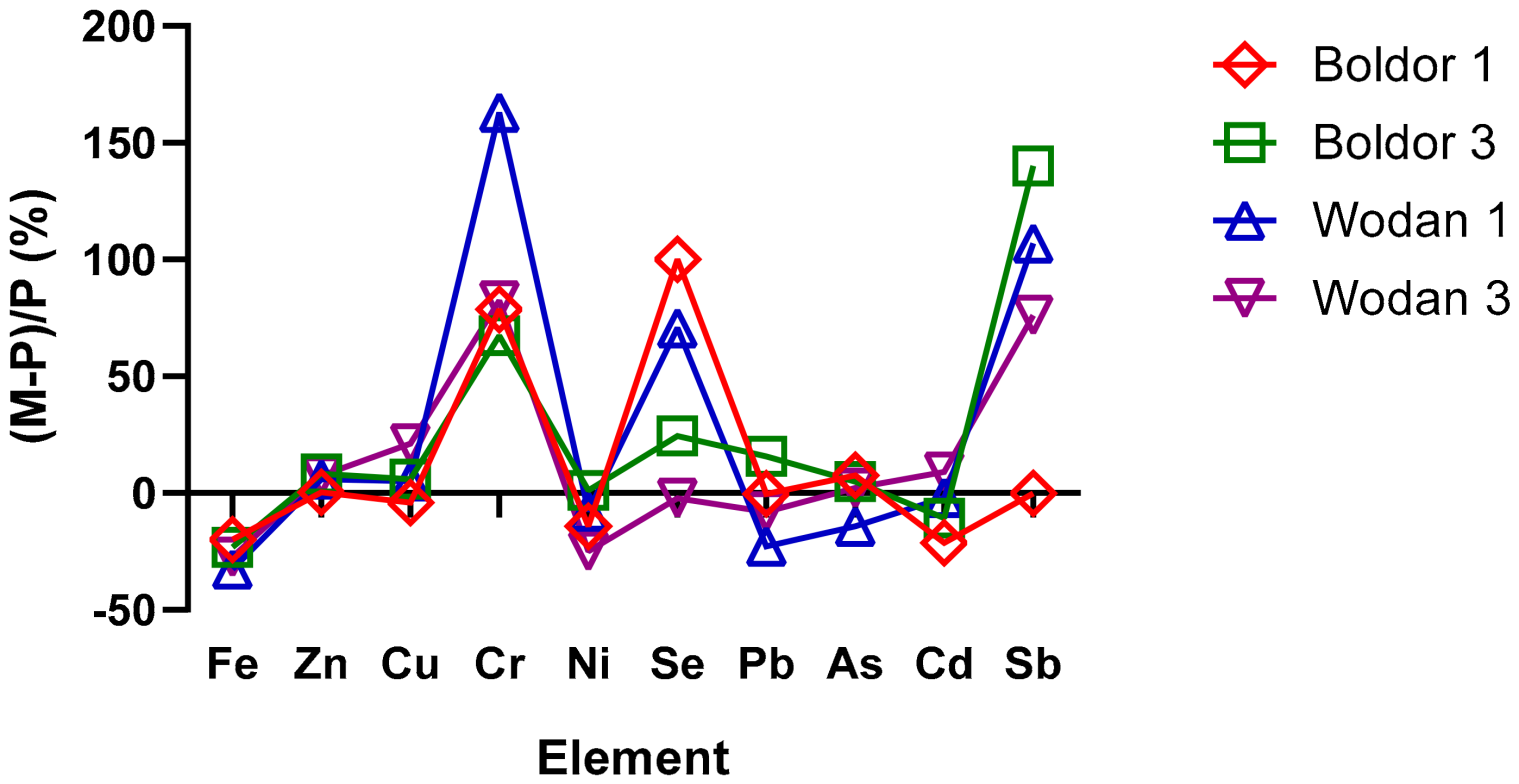
Relative differences between measured (M) and predicted (P) concentrations, expressed as (M − P)/P (%), for Fe, Zn, Cu, Cr, Ni, Se, Pb, As, Cd, and Sb in diets supplemented with beetroot cultivars *Boldor* and *Wodan*. Dietary variants include *Boldor* 1, *Boldor* 3, *Wodan* 1, and *Wodan* 3, either without (level 1) or with (level 3) foliar application of the selenium-based plant growth stimulator. Positive values indicate higher measured than predicted concentrations, whereas negative values indicate lower measured values relative to predictions. Abbreviations: M, measured concentration; P, predicted concentration; Fe, iron; Zn, zinc; Cu, copper; Cr, chromium; Ni, nickel; Se, selenium; Pb, lead; As, arsenic; Cd, cadmium; Sb, antimony.

The comparison between predicted (P) and measured (M) values revealed element-specific discrepancies and clear differences between dietary groups (Figure 1; Table 3, Table 4 and Table 5 and Table S2).

The largest discrepancies were observed for Cr. As shown in Figure 1 and Table 3, measured Cr concentrations consistently exceeded predicted values in all dietary variants, with particularly pronounced differences in *Wodan* 1, for which the measured concentration was 2.047 mg/kg compared with a predicted concentration of 0.779 mg/kg. This is further supported by Table 5, which shows high ratios and the largest positive relative deviations for Cr. Notably, Table S5 indicates a high 4% beetroot component-to-basal diet ratio for Cr, especially in *Wodan* diets, indicating that the beetroot additive strongly contributed to its elevated levels and amplified discrepancies.

In contrast, Fe was consistently overestimated by the model, as measured values were lower than predicted across all diets, as shown in Table 3. This is reflected in M/P ratios below 1.0 (Table 3) and negative relative differences in Figure 1. Although Table S5 shows a substantial contribution of the beetroot component to Fe concentrations, particularly in Wodan diets, this contribution was not reflected in the measured values, suggesting limitations of the additive model or inaccuracies in the assumed composition of raw materials.

Arsenic (As, 2.87-fold), Cd (2.78-fold), and Pb (2.47-fold) showed consistently higher concentrations in *Wodan* diets compared to *Boldor* diets (Table 3), which is also reflected in the high W1/B1 and W3/B3 ratios in Table 5. Table S5 further demonstrates very high 4% beetroot component-to-basal diet ratios for these elements, indicating that the beetroot additive was a major source. Despite this effect, the discrepancies between predicted and measured values remained moderate, as shown in Figure 1, suggesting relatively uniform distribution and good model performance.

Antimony (Sb) exhibited considerable variability between predicted and measured values. Although its contribution from the dietary component was moderate (Table S5), large relative deviations were observed, particularly in *Boldor* 3 and *Wodan* 1 (Figure 1; Table 5), indicating inconsistent model performance and possible effects of sample heterogeneity.

Selenium (Se) showed moderate discrepancies between predicted and measured values, with some variability between dietary variants but no consistent pattern across cultivars (Table 3; Figure 1). This corresponds to its relatively low contribution from the dietary component (Table S5).

In contrast, Cu, Zn, and Ni showed close agreement between predicted and measured values, with M/P ratios close to 1.0 (Table 3), minimal variation in ratios (Table 5), and small relative differences (Figure 1). Their low 4% beetroot component-to-basal diet ratios (Table S5) indicate limited influence of the additive and stable distribution in the diets.

Overall, the magnitude and direction of discrepancies between predicted and measured values depended on both the element and dietary group and were strongly associated with the relative contribution of the dietary component. Elements with high 4% beetroot component-to-basal diet ratios (Cr, Pb, As, Cd) showed greater variability and stronger dependence on the additive, whereas elements with low ratios (Cu, Zn) remained stable and were more accurately predicted by the additive model.

## 4. Discussion

The use of different beetroot cultivars and foliar application of a selenium-based plant growth stimulator was expected to influence the elemental composition of the experimental diets. However, the observed variability was not associated with a consistent treatment-related pattern, suggesting that additional biological and methodological factors contributed to the final elemental profiles. Significant cultivar-dependent differences were observed for several trace elements, including some toxic elements, indicating that genotype may influence elemental uptake and accumulation in beetroot tissues. Consequently, cultivar selection may contribute to differences in the elemental composition of harvested roots, which is further supported by our recent in vivo study demonstrating distinct elemental profiles and physiological responses associated with the *Boldor* and *Wodan* cultivars [32]. No statistically significant effect of the selenium-based plant growth stimulator was detected in the final experimental diets, as treatment effects were generally non-significant and no clear interaction between cultivar and treatment was observed.

In the present study, the content of microelements and toxic elements in final male WKY rat diets supplemented with 4% beetroot was evaluated by comparing predicted values, calculated using an additive model, with experimentally measured values. The results revealed substantial discrepancies between theoretical and analytical data, indicating the influence of raw material variability, technological factors, and inherent limitations of the applied model. Such inconsistencies between theoretical and measured compositions have also been reported for plant-derived materials and food systems [21,22,33], as well as in studies highlighting long-term variability in mineral composition of plant foods [23]. The contribution of the dietary component played a key role in shaping element distribution, particularly for elements with high 4% beetroot component-to-basal diet ratios.

The largest deviations between predicted and measured values were observed for Cr, particularly in the *Wodan* 1 diet, where measured concentrations were several times higher than predicted. Similarly, considerable variability was found for Sb, especially in *Boldor* 3 and *Wodan* diets. As shown in Table S5, Cr exhibited a high 4% beetroot component-to-basal diet ratio, particularly in *Wodan* diets, indicating that the beetroot additive was a major source of this element and strongly influenced its final concentration. This reflected the markedly higher concentrations of these elements in the *Wodan* cultivar compared with *Boldor* (Table S3). This is consistent with previous findings demonstrating that the mineral composition of beetroot varies depending on cultivar and environmental conditions [21,33]. In contrast, Sb showed only a moderate contribution from the additive, suggesting that its variability cannot be explained solely by the component input and may also reflect sample heterogeneity or other unaccounted sources.

All diets were processed in the form of dried powder, a system known to be prone to incomplete mixing and secondary segregation during handling, transport, and storage. Differences in particle size, shape, and density between dietary components can lead to stratification, resulting in localized variation in chemical composition. In particular, the 4% beetroot additive may not have been evenly distributed throughout the mixture or may have migrated within the bulk material. Consequently, the analytical sample may not have been fully representative of the entire batch, leading to over- or underestimation of certain trace elements, especially those present at low concentrations. Similar issues related to particle segregation and mixing efficiency have been widely reported in feed manufacturing and powder technology [34,35].

Previous reviews have highlighted that accurate determination of trace elements in complex matrices depends not only on instrumental sensitivity but also on sample preparation, digestion efficiency, matrix effects and quality-control procedures. These factors may contribute to variability observed between theoretical and experimentally determined concentrations [31].

In contrast, Fe concentrations were consistently lower in measured values than predicted across all diets, indicating systematic overestimation by the additive model. Iron plays a central role in metabolism and immune function, and its bioavailability may be affected by dietary interactions [36,37]. Although Table S5 indicates a relatively high contribution of the beetroot component to Fe levels, particularly in *Wodan* diets, this contribution was not reflected in the measured values. This suggests that the discrepancies are more likely attributable to limitations of the additive model or inaccuracies in the assumed composition of raw materials, rather than to sample heterogeneity. Additionally, the additive model does not account for interactions among dietary components, which may contribute to the observed discrepancies [38]. This interpretation is supported by studies demonstrating that iron bioavailability and retention depend on complex dietary interactions [39,40].

A noteworthy finding was the markedly higher concentrations of Pb, As, and Cd in *Wodan* diets than in *Boldor* diets. Their high 4% beetroot component-to-basal diet ratios (Table S5) indicate that the beetroot additive was the dominant source of these elements in the final diets. In contrast to Cr and Sb, predicted and measured concentrations showed relatively good agreement, suggesting more uniform distribution within the dietary mixtures. The observed cultivar-related differences are consistent with previous reports showing that beetroot and other crop plants can accumulate toxic elements from soil, including Pb, Cd, and As, and that their concentrations may be influenced by cultivation conditions and fertilization practices [41,42,43,44,45,46,47].

Although the toxicity of arsenic and chromium depends on their chemical speciation, total element concentrations were determined in the present study as an initial screening approach for evaluating the additive model. In beetroot-based diets, chromium is expected to occur predominantly as Cr(III), although chromium speciation was not determined in the present study, as Cr(VI) is generally unstable under organic-rich, reducing, and mildly acidic conditions. Chromium speciation was beyond the scope of the present study. Therefore, the determination of total chromium provided an appropriate basis for evaluating the predictive performance of the additive model. Similar cultivar-dependent trends were observed for Fe, Cr, and Sb, all of which occurred at higher concentrations in diets prepared from the *Wodan* cultivar.

The measured concentrations of potentially toxic elements were also compared with the maximum permitted levels established in Directive 2002/32/EC on undesirable substances in animal feed. For complete feed, the maximum permitted concentrations, expressed relative to a feed with a moisture content of 12%, are 2 mg/kg for As, 0.5 mg/kg for Cd, and 5 mg/kg for Pb. The highest concentrations measured in the experimental diets were 0.070, 0.013, and 0.087 mg/kg dry matter for As, Cd, and Pb, respectively. After conversion to a 12% moisture basis, the corresponding concentrations were 0.0616, 0.0114, and 0.0766 mg/kg. Therefore, all experimental diets contained As, Cd, and Pb at concentrations substantially below the applicable maximum levels, indicating that the observed differences between diets did not represent a feed safety concern.

In contrast, Cu and Zn exhibited minor differences between predicted and measured values, indicating stable behavior and suggesting that the additive model performs adequately for these elements. This is consistent with their low 4% beetroot component-to-basal diet ratios (Table S5), indicating limited influence of the additive and more uniform distribution within the diets. Similar stability of selected essential elements in vegetables has been reported in the literature [48].

Importantly, as all diets were analyzed in dried form, differences in moisture content and associated concentration effects can be excluded as major contributors to the observed discrepancies. Therefore, the deviations between predicted and measured values may be attributed primarily to raw material variability, potential contamination, limitations of the additive model, and insufficient mixing and/or segregation within the powdered diets. Such variability among plant-derived materials has been widely documented and is consistent with previous reports on beetroot mineral composition variability (see Introduction).

Previous studies have also investigated beet leaves as a potential source of iron and other micronutrients for dietary supplementation. In several cases, beet leaves exhibited higher concentrations of selected minerals than storage roots. However, the present study focused exclusively on beetroot-derived dietary components because roots represent the commercially relevant fraction most commonly used in food and feed applications. Future studies comparing roots and leaves within the same cultivation system could provide additional insight into the contribution of different beet tissues to dietary trace element intake. Another limitation of the present study is the use of a simple linear additive model as the predictive benchmark. The model assumes complete homogeneity of the diet, constant elemental composition of all raw materials, and proportional contribution of each component to the final mixture. In practice, these assumptions are rarely fully satisfied. Variability in raw material composition, differences in particle size and density, incomplete mixing, and analytical uncertainty may all contribute to deviations between theoretical and measured concentrations. Furthermore, the model does not account for uncertainty propagation from individual components to the final prediction, which may be particularly relevant for elements present at low concentrations or characterized by high component-to-basal diet ratios. Consequently, the predictive performance of the additive model is expected to vary among elements depending on their concentration range and distribution within the diet matrix. The observed discrepancies for chromium, iron, and antimony, together with the moderate variability observed for selenium, indicate that simple additive approaches may have limited applicability for accurately estimating trace element concentrations in complex dietary systems. Future studies should consider incorporating uncertainty analysis, sensitivity assessment, and more advanced modelling approaches to improve prediction reliability. Therefore, the additive model should be regarded primarily as a useful theoretical reference rather than an exact predictor of elemental composition in heterogeneous diet matrices. A limitation of the present study is the lack of formal error propagation analysis for the additive model. Therefore, the cumulative impact of analytical uncertainty and raw material variability on prediction accuracy could not be quantified and should be addressed in future investigations. Although powdered diets may be susceptible to particle segregation during handling and storage, the homogeneity of the prepared diets was not experimentally evaluated in the present study. Therefore, sample heterogeneity cannot be considered a confirmed explanation for the observed discrepancies and should be regarded only as a potential limitation.

The present study does not evaluate biological toxicity itself; rather, it addresses the analytical reliability of exposure assessment. Because accurate exposure characterization is a prerequisite for valid toxicological interpretation, verification of elemental concentrations in experimental diets should be considered an integral part of toxicological study design.

## 5. Conclusions

In summary, the results demonstrate that while additive models provide a useful approximation of mineral composition, their predictive accuracy is limited, particularly for elements strongly influenced by the relative contribution of the dietary component and by variability associated with sample preparation. Significant differences in elemental composition were primarily associated with beetroot cultivar, whereas foliar application of the selenium-based plant growth stimulator did not significantly affect the concentrations of the analyzed microelements and toxic elements. These findings highlight the importance of thorough mixing, prevention of segregation, representative sampling, and strict quality control of plant-based raw materials in feed formulation. From a toxicological perspective, reliance solely on theoretical additive calculations may result in inaccurate estimation of exposure to toxic elements. Therefore, analytical verification of experimental diets should be regarded as an essential quality-control step before conducting dietary toxicology studies.

## Figures and Tables

**Table 1:** Dietary composition (%).

Ingredient	Control (%)	*Wodan* and *Boldor* (%)
Casein	8.0	8.0
Methionine	0.3	0.3
Rapeseed oil	2.0	2.0
Lard	10	10
Sucrose	11	11
Cellulose	5.0	5.0
Mineral mixture	3.5	3.5
Vitamin mixture	1.0	1.0
Choline chloride	0.2	0.2
Beetroot	0	4.0
Corn starch	59	55
Total	100	100

Mineral mixture (%): Iron citrate [16.7% Fe]—31.0; Zinc carbonate (ZnCO_3_) [56% Zn]—4.5; Manganese carbonate (MnCO_3_) [44.4% Mn]—23.4; Copper carbonate (CuCO_3_) [55.5% Cu]—1.85; Potassium iodide (KI)—0.04; Citric acid—39.21. Although the estimated consumption was approximately 8 kg per group over the 45-day feeding period, 10 kg of diet was prepared per group to ensure sufficient feed availability throughout the study and to account for feed losses (e.g., spillage and uneaten feed).

**Table 2:** ICP-MS Operating Parameters Applied for Elemental Determination.

Parameter	Operating Condition
ICP-MS system	ICP-MS 7700×
Cone material	Ni
RF power (W)	1550
Nebulizer gas flow (L/min)	0.98
Auxiliary gas flow (L/min)	0.90
Plasma gas flow (L/min)	15.50
Peristaltic pump speed (rps)	0.10
He flow (mL/min)	6.0 for Cu and Zn; 4.0 for Cr, Fe, Ni, Sb, and Se; 3.0 for As, Cd, and Pb
Selected isotopes	75As, 111Cd, 52Cr, 65Cu, 56Fe, 60Ni, 123Sb, 82Se, 208Pb, 68Zn
Integration time per m/z (s)	0.1
Number of replicates	3
Sweeps per replicate	100

The selenium signal was corrected using the following expression: Mc (82) = 1.0000 × M(82) − 1.0078 × M(83) where Mc(82) is the corrected intensity at m/z 82, while M(82) and M(83) correspond to the measured intensities at m/z 82 and 83, respectively.

**Table 3:** Microelements and toxic elements in experimental diets (mg/kg DW).

	Boldor 1			Boldor 3			Wodan 1			Wodan 3		
	Mean	SD	M/P	Mean	SD	M/P	Mean	SD	M/P	Mean	SD	M/P
Fe	73.97	0.53	0.80	73.88	12.22	0.77	127.2	0.88	0.67	128.4	1.41	0.73
Zn	34.03	0.18	1.00	36.79	6.37	1.08	36.91	0.34	1.06	37.28	0.10	1.07
Cu	5.570	0.08	0.96	6.124	1.05	1.06	6.138	0.04	1.05	7.083	0.05	1.21
Cr	0.811	0.01	1.79	0.807	0.13	1.67	2.047	0.03	2.63	1.789	0.02	1.82
Ni	0.550	0.01	0.86	0.651	0.02	1.01	0.655	0.05	0.92	0.530	0.03	0.75
Se	0.173	0.041	2.01	0.108	0.032	1.24	0.153	0.029	1.72	0.086	0.021	0.98
Pb	0.034	0.002	1.00	0.034	0.013	1.17	0.081	0.002	0.78	0.087	0.002	0.93
As	0.022	0.005	1.05	0.024	0.006	1.09	0.062	0.001	0.86	0.070	0.012	1.03
Cd	0.004	0.0009	0.80	0.005	0.0014	1.00	0.012	0.0011	1.00	0.013	0.0035	1.08
Sb	0.002	0.0002	1.00	0.005	0.0018	2.50	0.006	0.0019	2.00	0.005	0.0013	1.67

Measured elemental concentrations are presented as mean ± SD together with the measured-to-predicted ratio (M/P). Predicted concentrations were calculated using the additive model P = 0.96 × C _basal diet_ + 0.04 × C _beetroot component_. Both C _basal diet_ and C _beetroot component_ were determined experimentally using ICP-MS. Dietary treatments included the *Boldor* and *Wodan* cultivars grown either without (level 1) or with (level 3) foliar application of the selenium-based plant growth stimulator. *p*-values for the effects of cultivar, selenium treatment, and their interaction were obtained using two-way ANOVA; Statistical significance was accepted at *p* < 0.05. Values are based on four independent biological replicates (*n* = 4), each analyzed in triplicate (*m* = 3).

**Table 4:** Two-way ANOVA of the effects of cultivar, selenium treatment, and their interaction on elemental concentrations in experimental diets.

	Cultivar (A)	Selenium (B)	Interaction (A × B)
Fe	<0.0001	0.4687	0.3325
Zn	0.0408	0.0797	0.5243
Cu	0.0361	0.0689	0.0772
Cr	<0.0001	0.0601	0.1101
Ni	0.0010	0.0715	0.0902
Se	0.2873	0.0739	0.6787
Pb	<0.0001	0.0931	0.1145
As	<0.0001	0.1202	0.4679
Cd	0.0001	0.0976	0.4959
Sb	0.0359	0.2187	0.0825

*p*-values from the two-way ANOVA evaluating the effects of cultivar (A), selenium treatment (B); level 1 = without and level 3 = with foliar application of the selenium-based plant growth stimulator, and their interaction (A × B). Statistical significance was accepted at *p* < 0.05. Values are based on four independent biological replicates (*n* = 4), each analyzed in triplicate (*m* = 3).

**Table 5:** Ratios of microelement and toxic element concentrations in experimental diets (B3/B1, W3/W1, W1/B1, W3/B3).

Element	P				M			
	B3/B1	W3/W1	W1/B1	W3/B3	B3/B1	W3/W1	W1/B1	W3/B3
Fe	1.0	0.9	2.0	1.8	1.0	1.0	1.7	1.7
Zn	1.0	1.0	1.0	1.0	1.1	1.0	1.1	1.0
Cu	1.0	1.0	1.0	1.0	1.1	1.2	1.1	1.2
Cr	1.1	0.9	1.7	1.5	1.0	0.6	2.5	1.6
Ni	1.0	1.0	1.1	1.1	1.2	0.8	1.2	0.8
Se	1.0	1.0	1.0	1.0	0.6	0.6	0.9	0.8
Pb	0.9	0.9	3.1	3.2	1.0	1.1	2.4	2.6
As	1.0	0.9	3.4	3.1	1.1	1.1	2.8	2.9
Cd	1.0	1.0	2.4	2.4	1.3	1.1	3.0	2.6
Sb	1.0	1.0	1.5	1.5	2.5	0.8	3.0	1.0

*C _basal diet_* and *C _beetroot component_* were determined experimentally. P—predicted concentrations calculated using the additive model (*P* = 0.96 × *C _basal diet_* + 0.04 × *C _beetroot component_*); M—measured concentrations determined using ICP-MS. B and W denote beetroot cultivars *Boldor* and *Wodan*, respectively. The numbers 1 and 3 indicate diets without (level 1) and with (level 3) foliar application of the selenium–based plant growth stimulator. Ratios (B3/B1, W3/W1, W1/B1, W3/B3) represent relative changes in element concentrations between the respective dietary variants.

## Data Availability

The original contributions presented in this study are included in the article/Supplementary Material. Further inquiries can be directed to the corresponding author.

## Supplementary Materials

The following supporting information can be downloaded at https://www.mdpi.com/article/10.3390/toxics14080721/s1, Table S1: Predicted elemental concentrations in experimental diets calculated using the additive model; Table S2: Concentrations of selected trace elements in the basal control diet; Table S3: Elemental composition of beetroot roots; Table S4: Certified and measured concentrations in NIST SRM 1570a Spinach Leaves; Table S5: Relative contribution of the 4% beetroot inclusion.

## References

[1] Zhao D., Wang P., Zhao F.-J. (2024). Toxic metals and metalloids in food: Current status, health risks, and mitigation strategies. Curr. Environ. Health Rep..

[2] Srivastava R., Singh Y., White J.C., Dhankher O.P. (2024). Mitigating toxic metals contamination in foods: Bridging knowledge gaps for addressing food safety. Trends Food Sci. Technol..

[3] Kitala K., Tanski D., Godlewski J., Krajewska-Włodarczyk M., Gromadziński L., Majewski M. (2023). Copper and zinc particles as regulators of cardiovascular system function—A review. Nutrients.

[4] Kitala-Tańska K., Hanć A., Juśkiewicz J., Majewski M. (2024). Prolonged copper supplementation modified minerals in the kidney, liver and blood, and potentiated oxidative stress and vasodilation of isolated aortic rings in young wistar rats. Nutrients.

[5] Majewski M., Gromadziński L., Cholewińska E., Ognik K., Fotschki B., Juśkiewicz J. (2023). The interaction of dietary pectin, inulin, and psyllium with copper nanoparticle induced changes to the cardiovascular system. Nutrients.

[6] Kitala-Tańska K., Socha K., Juśkiewicz J., Krajewska-Włodarczyk M., Majewski M. (2024). The effect of an elevated dietary copper level on the vascular contractility and oxidative stress in middle-aged rats. Nutrients.

[7] Borkowska-Sztachańska M., Thoene M., Socha K., Juśkiewicz J., Majewski M.S. (2024). Decreased vascular contraction and changes in oxidative state in middle-aged wistar rats after exposure to increased levels of dietary zinc. Toxicol. Appl. Pharmacol..

[8] Kaur N., Singh J., Sharma N.R., Natt S.K., Mohan A., Malik T., Girdhar M. (2025). Heavy metal contamination in wastewater-irrigated vegetables: Assessing food safety challenges in developing Asian countries. Environ. Sci. Process. Impacts.

[9] Pourret O., Hursthouse A. (2019). It’s Time to Replace the Term “Heavy Metals” with “Potentially Toxic Elements” When Reporting Environmental Research. Int. J. Environ. Res. Public Health.

[10] Zhang X., Barceló D., Clougherty R.J., Gao B., Harms H., Tefsen B., Vithanage M., Wang H., Wang Z., Wells M. (2022). “Potentially Toxic Element”—Something that Means Everything Means Nothing. Environ. Sci. Technol..

[11] Stoica F., Râpeanu G., Rațu R.N., Stănciuc N., Croitoru C., Țopa D., Jităreanu G. (2025). Red beetroot and its by-products: A comprehensive review of phytochemicals, extraction methods, health benefits, and applications. Agriculture.

[12] Erdal İ., Yıldız Y., Yalçın S.S., Yirün A., Çakır D.A., Erkekoğlu P. (2024). Heavy metal and trace element status and dietary determinants in children with phenylketonuria. Nutrients.

[13] Balali-Mood M., Naseri K., Tahergorabi Z., Khazdair M.R., Sadeghi M. (2021). Toxic mechanisms of five heavy metals: Mercury, lead, chromium, cadmium, and arsenic. Front. Pharmacol..

[14] Clifford T., Howatson G., West D.J., Stevenson E.J. (2015). The potential benefits of red beetroot supplementation in health and disease. Nutrients.

[15] Nikan M., Manayi A., Nabavi S.M., Silva A.S. (2019). *Beta vulgaris* L.. Nonvitamin and Nonmineral Nutritional Supplements.

[16] Chhikara N., Kushwaha K., Sharma P., Gat Y., Panghal A. (2019). Bioactive compounds of beetroot and utilization in food processing industry: A critical review. Food Chem..

[17] Ninfali P., Angelino D. (2013). Nutritional and functional potential of beta vulgaris cicla and rubra. Fitoterapia.

[18] Odoh U.E., Okoro E.C. (2013). Quantitative phytochemical, proximate/nutritive composition analysis of Beta vulgaris Linnaeus (Chenopodiaceae). Int. J. Curr. Res..

[19] Sharma K.D., Karki S., Thakur N.S., Attri S. (2012). Chemical composition, functional properties and processing of carrot—A review. J. Food Sci. Technol..

[20] Székely D., Furulyás D., Stéger-Máté M. (2019). Investigation of mineral and vitamin c contents in different parts of beetroots (*Beta vulgaris* L.). Not. Bot. Horti Agrobot. Cluj-Napoca.

[21] Ndunge G., Njoroge Kariuki D., Mangala J., Gatari M. (2020). Analysis of beetroot bulbs (*Beta vulgaris*) from selected geographical regions in Kenya: Essential nutritional elements contents. J. Food Nutr. Sci..

[22] Petek M., Toth N., Pecina M., Karažija T., Lazarević B., Palčić I., Veres S., Ćustić M.H. (2019). Beetroot mineral composition affected by mineral and organic fertilization. PLoS ONE.

[23] Ekholm P., Reinivuo H., Mattila P., Pakkala H., Koponen J., Happonen A., Hellström J., Ovaskainen M.L. (2007). Changes in the mineral and trace element contents of cereals, fruits and vegetables in Finland. J. Food Compos. Anal..

[24] Brzezińska-Rojek J., Rutkowska M., Brzezicha J., Konieczka P., Prokopowicz M., Grembecka M. (2022). Mineral composition of dietary supplements-analytical and chemometric approach. Nutrients.

[25] Scutarașu E.C., Trincă L.C. (2023). Heavy metals in foods and beverages: Global situation, health risks and reduction methods. Foods.

[26] Brzezińska-Rojek J., Rutkowska M., Ośko J., Konieczka P., Prokopowicz M., Grembecka M. (2024). Evaluation of the safety and potential benefits of beetroot-based dietary supplements according to their elemental composition. Biol. Trace Elem. Res..

[27] Peralta-Videa J.R., Lopez M.L., Narayan M., Saupe G., Gardea-Torresdey J. (2009). The biochemistry of environmental heavy metal uptake by plants: Implications for the food chain. Int. J. Biochem. Cell Biol..

[28] Schryvers S., Du Laing G., Lachat C., Jacxsens L. (2026). Risk ranking of toxic trace elements in animal- and plant-based protein sources under dietary protein transition scenarios. Food Control.

[29] White P.J., Broadley M.R. (2005). Biofortifying crops with essential mineral elements. Trends Plant Sci..

[30] Hasanuzzaman M., Bhuyan M.B., Raza A., Hawrylak-Nowak B., Matraszek-Gawron R., Al Mahmud J., Nahar K., Fujita M. (2020). Selenium in plants: Boon or bane?. Environ. Exp. Bot..

[31] Smichowski P., Londonio A. (2018). The role of analytical techniques in the determination of metals and metalloids in dietary supplements: A review. Microchem. J..

[32] Majewski M.S., Hanć A., Krajewska-Włodarczyk M., Majkowska-Gadomska J., Francke A. (2026). Preliminary Assessment of Red Beetroot Supplementation and Cultivar Effects in Low-Protein-Fed WKY Rats. Nutrients.

[33] Wruss J., Waldenberger G., Huemer S., Uygun P., Lanzerstorfer P., Müller U., Höglinger O., Weghuber J. (2015). Compositional characteristics of commercial beetroot products and beetroot juice prepared from seven beetroot varieties grown in Upper Austria. J. Food Compos. Anal..

[34] Behnke K.C. (1996). Feed manufacturing technology: Current issues and challenges. Anim. Feed Sci. Technol..

[35] McCabe W.L., Smith J.C., Harriott P. (2005). Unit Operations of Chemical Engineering.

[36] Briguglio M., Hrelia S., Malaguti M., Lombardi G., Riso P., Porrini M., Perazzo P., Banfi G. (2020). The central role of iron in human nutrition: From folk to contemporary medicine. Nutrients.

[37] Oppenheimer S.J. (2001). Iron and its relation to immunity and infectious disease. J. Nutr..

[38] Suttle N.F. (2010). Mineral Nutrition of Livestock.

[39] Fairweather-Tait S.J., Teucher B. (2002). Iron and calcium bioavailability of fortified foods and dietary supplements. Nutr. Rev..

[40] Santiago P. (2012). Ferrous versus ferric oral iron formulations for the treatment of iron deficiency: A clinical overview. Sci. World J..

[41] Kabata-Pendias A., Mukherjee A.B. (2007). Trace Elements from Soil to Human.

[42] Adams S.N. (1961). The Effect of sodium and potassium fertilizer on the mineral composition of sugar beet. J. Agric. Sci..

[43] Ćwieląg-Drabek M., Piekut A., Gut K., Grabowski M. (2020). Risk of cadmium, lead and zinc exposure from consumption of vegetables produced in areas with mining and smelting past. Sci. Rep..

[44] Magro F.O., da Silva E.G., Takata W.H.S., Cardoso A.I.I., Fernandes D.M., Evangelista R.M. (2015). Organic compost and potassium top dressing fertilization on production and quality of beetroot. Aust. J. Crop Sci..

[45] Gzyl J. (1990). Lead and cadmium contamination of soil and vegetables in the Upper Silesia region of Poland. Sci. Total Environ..

[46] Sękara A., Poniedziałek M., Ciura J., Jędrszczyk E. (2005). Cadmium and lead accumulation and distribution in the organs of nine crops: Implications for phytoremediation. Pol. J. Environ. Stud..

[47] Pizarro I., Gómez-Gómez M., León J., Román D., Palacios M.A. (2016). Bioaccessibility and arsenic speciation in carrots, beets and quinoa from a contaminated area of Chile. Sci. Total Environ..

[48] Grembecka M., Szefer P., Dybek K., Gurzyńska A. (2008). Ocena zawartości wybranych biopierwiastków w warzywach. Rocz. Panstw. Zakl. Hig..

