# Supplementary material for: Discrepancies Between Predicted and Measured Microelements and Toxic Elements in Beetroot Supplemented Diets
Source: Toxics. 2026 Aug 14;14(8):721. doi: 10.3390/toxics14080721 (PMC13517700; doi:10.3390/toxics14080721)
Supplement: Supplementary file 1 [file toxics-14-00721-s001.zip › toxics-4407308-supplementary.pdf]

Table S1. Predicted elemental concentrations in experimental diets calculated using the additive model (mg/kg dry matter).

	**Boldor 1**		**Boldor 3**		**Wodan 1**		**Wodan 3**	
	**mean**	**SD**	**mean**	**SD**	**mean**	**SD**	**mean**	**SD**
**Fe**	92.47	1.86	96.27	1.84	188.72	3.91	176.75	2.37
**Zn**	33.94	0.74	33.95	0.74	34.82	0.74	34.70	0.74
**Cu**	5.806	0.11	5.782	0.11	5.846	0.11	5.851	0.11
**Cr**	0.454	0.01	0.482	0.01	0.779	0.02	0.707	0.01
**Ni**	0.642	0.02	0.643	0.02	0.711	0.02	0.705	0.02
**Se**	0.086	0.008	0.087	0.008	0.089	0.009	0.088	0.009
**Pb**	0.026	0.004	0.029	0.005	0.104	0.005	0.094	0.003
**As**	0.021	0.001	0.022	0.001	0.072	0.003	0.068	0.002
**Cd**	0.005	0.0002	0.005	0.0002	0.012	0.0003	0.012	0.0002
**Sb**	0.002	0.0008	0.002	0.0008	0.003	0.0008	0.003	0.0008

Predicted concentrations were calculated using the additive model: $P = 0.96 \times C_{\text{basal diet}} + 0.04 \times C_{\text{beetroot component}}$, where the coefficients represent the proportional contributions of the basal diet (96%) and beetroot component (4%), respectively. Both component concentrations were determined experimentally using ICP-MS. Values of C basal diet and C beetroot are presented in Tables S2 and S3, respectively. The uncertainty (SD) of the predicted concentrations was estimated by standard error propagation using the standard deviations calculated across four independent biological replicates. Each biological replicate was analyzed in triplicate by ICP-MS, and the mean of the three technical determinations was used for statistical calculations.

Table S2. Concentrations of selected trace elements in the basal (control) diet (mg/kg dry matter).

**Element**	**Control diet (mean ± SD) [mg/kg]**
**Fe**	71.04 ± 1.86
**Zn**	34.28 ± 0.78
**Cu**	5.70 ± 0.12
**Cr**	0.381 ± 0.015
**Ni**	0.642 ± 0.021
**Se**	0.0857 ± 0.0089
**Pb**	0.0084 ± 0.0036
**As**	0.0070 ± 0.0014
**Cd**	0.00281 ± 0.00018
**Sb**	0.00186 ± 0.00087

Values are expressed as mean ± standard deviation (SD) of four independent biological replicates (n = 4), each analyzed in triplicate (m = 3). Element concentrations were determined by ICP-MS and are reported on a dry matter basis (mg/kg DM).

Table S3. Elemental composition of beetroot roots (mg/kg dry matter).

Element	Boldor 1	Boldor 3	Wodan 1	Wodan 3	Cultivar (A)	Selenium-based treatment (B)	Interaction (A × B)
**Fe**	606.85 ± 12.32	701.73 ± 10.31	3013.09 ± 86.92	2713.72 ± 38.69	< 0.001	0.880	0.860
**Zn**	25.82 ± 0.34	26.03 ± 0.22	47.83 ± 1.85	44.75 ± 0.50	0.043	0.420	0.536
**Cu**	8.42 ± 0.21	7.82 ± 0.13	9.44 ± 0.27	9.55 ± 0.15	0.005	0.079	0.022
**Cr**	2.20 ± 0.03	2.92 ± 0.05	10.33 ± 0.23	8.54 ± 0.04	< 0.0001	0.089	< 0.0001
**Ni**	0.62 ± 0.02	0.65 ± 0.02	2.37 ± 0.07	2.20 ± 0.03	0.001	0.075	0.090
**Se**	0.104 ± 0.016	0.107 ± 0.024	0.179 ± 0.041	0.147 ± 0.038	0.018	0.287	0.969
**Pb**	0.46 ± 0.04	0.53 ± 0.0005	2.41 ± 0.08	2.15 ± 0.01	0.004	0.093	0.092
**As**	0.35 ± 0.01	0.39 ± 0.0006	1.64 ± 0.07	1.54 ± 0.03	< 0.0001	0.308	0.468
**Cd**	0.066 ± 0.003	0.069 ± 0.002	0.239 ± 0.007	0.238 ± 0.005	0.0001	0.430	0.801
**Sb**	0.010 ± 0.003	0.012 ± 0.002	0.025 ± 0.003	0.023 ± 0.002	0.048	0.218	0.082

Values are presented as mean ± SD. Statistical significance was accepted at $p < 0.05$. Values are based on four independent biological replicates ($n = 4$), each analyzed in triplicate ($m = 3$). The effects of cultivar, selenium-based treatment, and their interaction. Values in the last three columns represent p-values obtained from two-way ANOVA.

Table S4. Certified and measured concentrations of trace elements in the certified reference material NIST SRM 1570a Spinach Leaves.

Element	Certified or reference value (mg/kg)	Measured value $\bar{x}$ ± SD (mg/kg)	Recovery (%)
**Fe**	—	236 ± 47	—
**Zn**	82.3 ± 3.9	77.3 ± 5.5	93.9
**Cu**	12.22 ± 0.86	12.2 ± 1.2	99.8
**Cr**	—	1.57 ± 0.35	—
**Ni**	2.142 ± 0.058	2.111 ± 0.064	98.6
**Se**	0.1152 ± 0.0043	0.1180 ± 0.0067	102.4
**Pb**	0.2*	0.198 ± 0.056	99.0
**As**	0.068 ± 0.012	0.072 ± 0.010	105.9
**Cd**	2.876 ± 0.058	2.866 ± 0.035	99.7
**Sb**	—	0.0251 ± 0.0014	—

Notes: Measured values are presented as mean ± SD ($n = 3$). Recovery was calculated as the measured value divided by the certified or reference value × 100. *Information value provided for reference only; —, no certified or information value available.

Table S5. Relative elemental contribution of the 4% beetroot component compared with the elemental content of the basal diet.

Element	Boldor 1	Boldor 3	Wodan 1	Wodan 3
**Fe**	0.34	0.40	1.70	1.53
**Zn**	0.03	0.03	0.06	0.05
**Cu**	0.06	0.06	0.07	0.07
**Cr**	0.23	0.31	1.08	0.90
**Ni**	0.04	0.04	0.15	0.14
**Se**	0.05	0.05	0.08	0.07
**Pb**	2.19	2.52	11.56	10.32
**As**	2.00	2.26	9.40	8.80
**Cd**	0.94	0.98	3.40	3.38
**Sb**	0.21	0.25	0.53	0.49

Values were calculated as: Contribution Index = $(0.04 \times C_{beetroot}) / C_{basal\ diet}$. The index expresses the amount of an element contributed by the 4% beetroot component relative to the nominal elemental concentration of the basal diet and is intended as a comparative indicator rather than a direct estimate of the elemental contribution in the final diet mixture. A value of 1 indicates equal nominal concentrations, whereas values >1 or <1 indicate proportionally higher or lower contributions. Elemental concentrations of the beetroot cultivars are presented in Table S3.
